# Body Uneasiness and Dissatisfaction Among Lesbian, Gay, Bisexual, and Heterosexual Persons

**DOI:** 10.1007/s13178-023-00805-3

**Published:** 2023-03-14

**Authors:** Laura Muzi, Nicola Nardelli, Gabriele Naticchioni, Claudia Mazzeschi, Roberto Baiocco, Vittorio Lingiardi

**Affiliations:** 1 Department of Philosophy, Social Sciences, Humanities and Education, University of Perugia, Piazza Ermini, 1, Perugia, 06123 Italy; 2 Mental Health Center, ASL Roma 1 UOC SM 2, Rome, Italy; 3 Department of Dynamic and Clinical Psychology, and Health Studies, Faculty of Medicine and Psychology, Sapienza University of Rome, Rome, Italy; 4 Department of Social and Developmental Psychology, Faculty of Medicine and Psychology, Sapienza University of Rome, Rome, Italy

**Keywords:** Body image, Body dissatisfaction, Sexual orientation identity, LGB persons, Body mass index, Objectification

## Abstract

**Introduction:**

While sexual minority people have been widely considered at risk for developing a range of body image concerns, evidence of body dissatisfaction and shame amongst LGB (lesbian, gay, and bisexual) individuals is mixed. This study investigated differences in body uneasiness, body dissatisfaction, and self-blaming/attacking attitudes between LGB and heterosexual individuals, as well as within LGB groups, while also examining the predictive role of body mass index (BMI).

**Methods:**

A sample of cisgender lesbian women (*n* = 163), gay men (*n* = 277), bisexual women (*n* = 135), bisexual men (*n* = 39), heterosexual women (*n* = 398), and heterosexual men (*n* = 219) completed an online survey assessing different aspects of body image between May and July 2020.

**Results:**

Gay and bisexual men reported greater body image disturbance and self-blaming attitudes relative to heterosexual men. In contrast, lesbian women reported lower body uneasiness than their bisexual and heterosexual counterparts, but greater self-hate. Moreover, lesbian and bisexual women showed more body dissatisfaction than gay men, and bisexual individuals reported more body uneasiness than individuals in other sexual minority subgroups. Higher BMI emerged as a significant predictor of body image concerns and dissatisfaction.

**Conclusions:**

Body image dimensions showed sexual identity–based differences. Determining the specific nuances of body image in LGB individuals can provide important information on potential risk factors that may impact mental health outcomes.

**Policy Implications:**

In-depth knowledge of body dissatisfaction and uneasiness in individuals with LGB identities may have critical implications for the development of personalized prevention and treatment strategies.

Perceptions, feelings, and thoughts about one’s body can have a significant impact on psychological and social health. Previous studies have outlined that *body image* is a multidimensional umbrella construct, in terms of both its assessment and its associations with related concepts (Banfield & McCabe, 2002; Cash et al., 2004). *Body image disturbance* (also referred to as a *negative body image*) refers primarily to negative experiences related to one’s body weight and shape (Grogan, 2006). It has cognitive-affective, perceptual, and behavioral components, including an excessive emphasis on body weight and/or shape on self-evaluation, poor body size perception accuracy, and repeated body checking (e.g., Glashouwer et al., 2020). While a positive body image has been found to relate to psychological resources such as high self-esteem, a negative body image has been associated with a variety of adverse health outcomes (e.g., Thompson, 2004), including disordered eating behaviors, depression, psychological distress, and poor quality of life (Alvy, 2013; Calzo et al., 2015; Pistella et al., 2019; Stice & Shaw, 2002).

The present study focused on the constructs of *body uneasiness* and *body dissatisfaction*, as two of the potential declinations that may accompany body image disturbance (Levitan et al., 2019). Body dissatisfaction reflects discontent about one’s physical appearance (Tatangelo et al., 2016). Body uneasiness, in turn, includes not only dissatisfaction with particular body parts, shapes, or functions, but also a general feeling of uneasiness relating to one’s body or weight, which can lead to avoidance behaviors, compulsive checking behaviors, and detachment and/or estrangement feelings towards the body (Cuzzolaro et al., 2006). The study also considered self-criticizing and self-hating attacking behavior towards the self, as these factors have been found to be associated with shame related to body image (e.g., Ferreira et al., 2019).

Recently, research has begun to explore these issues in social identity groups that have not previously been a focus of body image researchers, including sexual minority adults (Andersen & Swami, 2021). However, the relationship between body image disturbance, gender, and sexual orientation identity (hereinafter referred to as *sexual identity*) remains controversial. The population of sexual minority people—which includes, but is not limited to, lesbian women, gay men, and bisexual individuals—has been widely considered at risk for developing body dissatisfaction and concerns (Calzo et al., 2017; Dahlenburg et al., 2020; Goldhammer et al., 2019; Mason et al., 2018). Sexual minority individuals also report a greater frequency of disordered eating symptoms (including body image disturbance), relative to their heterosexual counterparts (French et al., 1996; Kamody et al., 2020; Laska et al., 2015; Shearer et al., 2015; Yean et al., 2013).

Assigned gender at birth may represent an additional variable of interest for body image disturbance, as men and women are likely to face different pressures to achieve an ideal body and different levels of weight stigma (Myers & Crowther, 2009). Men tend to strive for a muscular and lean body (Laghi et al., 2013; Schaefer & Thompson, 2018; Tiggemann et al., 2007), whereas women tend to strive for a thin body (e.g., Gordon et al., 2010). Furthermore, several studies have revealed significant differences within men, which are less pronounced in women (Dahlenburg et al., 2020). Of note, similar levels of body dissatisfaction and its correlates have been found in samples of women, regardless of sexual identity, and some investigations have noted that levels of body dissatisfaction in women tend to exceed those in men (Basabas et al., 2019; McGuinness & Taylor, 2016).

Despite increasing interest in the impact of body dissatisfaction and uneasiness in sexual minority individuals, research has primarily focused on gay men, and comparatively fewer studies have been devoted to lesbian women and bisexual individuals (Morrison et al., 2004). Evidence suggests that, compared to heterosexual men, gay men are significantly more likely to experience body image concerns, body dissatisfaction, and body image–related anxiety; to prioritize bodily appearance in appraisals of self-worth; and to strive for thinness (Calzo et al., 2015; Feldman & Meyer, 2007; Levesque & Vichesky, 2006; McClain & Peebles, 2016; Yean et al., 2013). Additionally, gay men with high body dissatisfaction and disordered eating symptoms have been found to have impaired psychological well-being (Gil, 2007; Levesque & Vichesky, 2006), characterized by high self-criticism, low self-esteem, and depressive symptoms (Chaney, 2008; Reilly & Rudd, 2006; Russell & Keel, 2002; Tiggemann et al., 2007; Yelland & Tiggemann, 2003).

Conversely, studies comparing sexual minority and heterosexual women have produced mixed results. Some research has found that, compared with heterosexual women, lesbian women tend to place less emphasis on physical attractiveness (Gettelman & Thompson, 1993; Siever, 1994) and are less likely to have a negative body image, body image concerns, and high body dissatisfaction (Dahlenburg et al., 2020; French et al., 1996; Polimeni et al., 2009; Strong et al., 2000). Furthermore, lesbian sexual identity has been found to predict a positive body image and fewer negative attitudes towards eating and weight (Owens et al., 2003). However, other studies have found no differences between lesbian and heterosexual women regarding body dissatisfaction (e.g., Beren et al., 1996). Two meta-analytic reviews provided evidence that sexual minority and heterosexual women may experience similar levels of body image concerns and body image disturbance. Furthermore, correlates of body image concerns (e.g., sociocultural pressure, high negative affect, low self-esteem) have been found to be generally similar among lesbian, bisexual, and heterosexual women (Mason et al., 2018; Morrison et al., 2004). Several studies have also found equivalent rates of disordered eating symptoms in lesbian, bisexual, and heterosexual women (Feldman & Meyer, 2007; Strong et al., 2000), though some have found a higher rate of such symptoms in heterosexual women (Lakkis et al., 1999; Siever, 1994). Sampling bias and other methodological limitations, such as the wide range of measures employed to evaluate negative body image, may have contributed to these contradictory findings.

Research has also shown that gay men, lesbian women, and heterosexual women tend to have higher levels of negative body image and body dissatisfaction than heterosexual men (Dahlenburg et al., 2020; Peplau et al., 2009). However, only limited research has compared lesbian women and gay men, with preliminary findings suggesting that lesbian women report greater levels of body image disturbance than gay men (Dahlenburg et al., 2020). Furthermore, there is a gap in the literature concerning body image disturbance in bisexual individuals, as very few studies have treated bisexual men and/or women as a specific group for analysis (Feldman & Meyer, 2007; Ryan et al., 2010). Despite the paucity of findings on this topic, current evidence suggests that bisexual individuals, and especially bisexual men, share some of the same body image concerns as other sexual minority men (Filiault et al., 2014).

Body weight and body mass index (BMI) are other under-researched variables in this field. Conceivably, these “objective” indices might influence the “subjective” internalization of weight bias and, in turn, bodily perception (Alvy, 2013). Sexual minority women have been found to report higher BMIs than heterosexual women, and some authors have suggested that they may be more likely to experience elevated rates of body size discrimination and harassment due to social stigmatization of overweight (Bowen et al., 2008; Conron et al., 2010; Johns et al., 2017). Furthermore, body weight and BMI have been found to predict body dissatisfaction in gay men (Frederick & Essayli, 2016; Peplau et al., 2009); however, the findings on lesbian and bisexual women are inconsistent.

Previous efforts to explore sexual identity–based differences in body image have mainly been grounded in sociocultural and objectification theories (Brewster et al., 2014). Sociocultural theories posit that idealized body images or norms differ between sexual minority and heterosexual men, with sexual minority men experiencing higher appearance pressure from peers, partners, the media, and the sexual minority community (Calzo et al., 2017; Jankowski et al., 2014). On the other hand, objectification theory (Fredrickson & Roberts, 1997), which was originally developed to account for disordered eating behaviors among women, suggests that societal appearance-related pressure and gender expectations may lead both men and women to view their body as a sexual object, provoking body surveillance behaviors and/or feelings of body-related shame (Gonzales & Blashill, 2021; Matsumoto & Rogers, 2020). Indeed, research has shown that, among gay men, increased sexual objectification may heighten body surveillance, body shame, and restricted eating (Martins et al., 2007; Tiggemann et al., 2007; Wiseman & Moradi, 2010). Among lesbian women, however, the rejection of heteronormative ideals and standards may protect against a negative body image and the development of harmful attitudes around eating and weight (Brown, 1987; Dahlenburg et al., 2020; Owens et al., 2003; Polimeni et al., 2009).

Drawing on the literature, the present study tested the following hypotheses:

- (a) There would be significant differences in levels of body image disturbance, body dissatisfaction, and self-criticism/self-hate between heterosexual and sexual minority men. More specifically, gay and bisexual men would show higher overall body uneasiness and its dimensions, as well as greater body dissatisfaction, feelings of inadequacy, and self-hate, compared to heterosexual men (e.g., Dahlenburg et al., 2020; Smith et al., 2011; Yean et al., 2013).
- (b) There would be no significant differences in levels of body uneasiness, body dissatisfaction, and self-criticism/self-hate between heterosexual and sexual minority women. Lesbian and bisexual women would show similar levels of body image disturbance and shame compared to heterosexual women (Mason et al., 2018; Morrison et al., 2004).
- (c) There would be significant differences between LGB (lesbian, gay, and bisexual) groups. In line with the scarce empirical evidence on sexual minority samples, lesbian women would show more body uneasiness and dissatisfaction compared to gay men (Dahlenburg et al., 2020; Markey et al., 2017).
- (d) BMI and body weight would predict levels of body uneasiness, body dissatisfaction, and self-criticism/self-hate in LGB people, even after controlling for age, assigned gender at birth, and sexual identity. Higher BMI and body weight would predict a negative body image and greater feelings of inadequacy and shame, especially in gay men (e.g., Frederick & Essayli, 2016; Peplau et al., 2009).

## Methods

### Participants and Procedure

Participants met the following inclusion criteria: (a) aged 18 years or older, (b) cisgender, and (c) of Italian nationality. They were asked to respond to an online survey, which was administered over a period of 2 months (May–July 2020). All questionnaires were delivered cross-sectionally through an online Google Forms survey, which was accessible via a designated link and required approximately 30–40 min for completion. All subjects who fulfilled the inclusion criteria were asked to participate in a study on self-image and body representation in LGB people. Information about the study was disseminated via organizations, online forums, listservs, and newsletters geared towards LGBTQ + individuals (by email and posts on websites and social networks), to reach a large number of subjects. All survey items had a forced response, to prevent missing data. Software was used to prevent a single individual from responding to the survey multiple times.

An initial sample of *N* = 1,248 participants completed the survey. Those who reported a different gender identity than that of their assigned gender (*n* = 4) and those who identified themselves as transgender woman/man (*n* = 3) or “other (please specify)” (*n* = 10) were not included, as such individuals may experience body image and body dissatisfaction uniquely (e.g., Pulice-Farrow et al., 2020; Witcomb et al., 2015). Out of the final study sample of *N* = 1,231 participants, 163 (13.2%) self-identified as lesbian women, 277 (22.5%) as gay men, 135 (11%) as bisexual women, 39 (3.2%) as bisexual men, 398 (32.3%) as heterosexual women, and 219 (17.8%) as heterosexual men.

Table 1 displays all descriptive characteristics of the study sample. In the overall sample, the mean age was 30.34 years (*SD* = 10.90). The average BMI was 23.61 kg/m^2^ and the mean body weight was 68.65 kg (*SD* = 15.11). All study subjects were White and Italian. The majority of respondents had a high school diploma (*N* = 440, 35.7%) or bachelor’s degree (*N* = 364, 29.6%). A slightly lower percentage of participants (*N* = 262, 21.5%) had a master’s degree, and 127 (10.3%) had a doctoral degree. Only 35 (2.8%) participants had a secondary school degree. Most participants were single (*N* = 583, 47.3%) or in a stable relationship (*N* = 508, 41.3%); a minor percentage of respondents were married (*N* = 113, 9.2%) or separated/divorced (*N* = 25, 2.1%). Only 2 participants (0.2%) were widowed. In terms of socioeconomic status, 690 (56.1%) declared a medium income level, 267 (21.7%) a low/medium income level, and 219 (17.8%) a medium/high income level; fewer participants declared a low (*N* = 41, 3.3%) or high (*N* = 14, 1.1%) income level.

**Table 1:** Descriptive Statistics (*N* = 1,231)

	LGB subgroups				Heterosexual subgroups		
Variable	Gay (*N* = 277) *M (SD)*	Lesbian (*N* = 163) *M (SD)*	Bisexual M (*N* = 39) *M (SD)*	Bisexual W (*N* = 135) *M (SD)*	HM^a^ (*N* = 219) *M (SD)*	HW^b^ (*N* = 398) *M (SD)*	Overall sample *M (SD)*
Age (years)	33.84 (12.29)	27.02 (7.27)	29.91 (8.84)	26.56 (7.01)	33.47 (11.34)	28.85 (10.12)	30.34 (10.90)
BMI (kg/m^2^)	23.62 (3.53)	23.46 (4.50)	22.71 (4.04)	23.32 (4.87)	25.21 (3.74)	22.94 (4.37)	23.61 (4.22)
Body weight	74.34 (12.87)	64.28 (13.26)	72.41 (14.54)	62.92 (15.04)	79.68 (13.91)	62.04 (12.81)	68.65 (15.11)
	*N* (%)	*N* (%)	*N* (%)	*N* (%)	*N* (%)	*N* (%)	*N* (%)
Marital status							
Single (never married)	183 (66.1)	66 (40.5)	22 (56.4)	62 (45.9)	87 (39.7)	163 (40.9)	583 (47.3)
Partnered	83 (29.9)	94 (57.6)	15 (38.5)	67 (49.6)	69 (31.5)	180 (45.2)	508 (41.3)
Married	10 (3.6)	2 (1.2)	2 (5.1)	3 (2.2)	54 (24.6)	42 (10.5)	113 (9.2)
Divorced/separated	1 (0.4)	1 (0.6)	0 (0)	3 (2.2)	9 (4.1)	11 (2.7)	25 (2.1)
Widowed	0 (0)	0 (0)	0 (0)	0 (0)	0 (0)	2 (0.5)	2 (0.2)
Highest education level							
Secondary school	4 (1.4)	5 (3.1)	2 (5.1)	3 (2.2)	16 (7.3)	5 (1.2)	35 (2.8)
High school	80 (28.9)	74 (45.4)	15 (38.4)	52 (38.5)	89 (40.6)	130 (32.6)	440 (35.7)
Bachelor degree	76 (27.4)	50 (30.7)	10 (25.6)	48 (35.5)	55 (25.1)	125 (31.4)	364 (29.6)
Master degree	76 (27.4)	26 (15.9)	7 (17.9)	26 (19.2)	40 (18.2)	90 (22.6)	265 (21.5)
Doctoral degree	41 (14.8)	8 (4.9)	5 (12.8)	6 (4.4)	19 (8.7)	48 (12.1)	127 (10.3)
Socioeconomic status							
Low	8 (2.8)	8 (4.9)	2 (5.1)	7 (5.2)	7 (3.2)	9 (2.3)	41 (3.3)
Low/medium	57 (20.6)	39 (23.9)	14 (35.9)	31(22.9)	50 (22.8)	76 (19.1)	267 (21.7)
Medium	151(54.5)	84 (51.5)	16 (41.1)	84 (62.2)	129 (58.9)	226 (56.8)	690 (56.1)
Medium/high	53 (19.1)	32 (19.6)	6 (15.3)	13 (9.6)	30 (13.7)	85 (21.6)	219 (17.8)
High	8 (2.8)	0 (0)	1 (2.5)	0 (0)	3 (1.4)	2 (0.5)	14 (1.1)

^a^Heterosexual men

^b^Heterosexual women

Significant differences emerged in terms of age (*F*[5, 1230] = 18.31, *p* < .001]), BMI (*F*[5, 1230] = 9.05, *p* < .001]), and body weight (*F*[5, 1230] = 68.02, *p* < .001]). Specifically, post-hoc analyses showed that gay and heterosexual men were older than lesbian, bisexual, and heterosexual women. Heterosexual men also had a higher BMI and body weight compared to all other groups, and gay men had a higher body weight than heterosexual, bisexual, and lesbian women.

The study protocol was reviewed and approved by the local research ethics committee and conducted in accordance with the 1964 Helsinki Declaration. Participation was voluntary. Prior to engaging in the survey, all participants provided electronic informed consent, indicating their understanding of both the study procedures and their right to cease their participation at any time, without penalty.

### Measures

#### Body Uneasiness Test – Form A

The Body Uneasiness Test – Form A (BUT-A; Cuzzolaro et al., 2006) is a 34-item self-report measure of several dimensions of body image in clinical and non-clinical populations, including intense fear of being or becoming fat (e.g., “I’m terrified of putting on weight”), body shape and/or weight dissatisfaction (e.g., “I feel I am fatter than others tell me”), avoidance (e.g., “The thought of some defects of my body torments me so much that it prevents me from being with others”), compulsive control behaviors (e.g., “If I begin to look at myself, I find it difficult to stop”), and detachment and estrangement feelings towards one’s body (e.g., “I have the sensation that my body does not belong to me”). Items are rated on a 6-point Likert scale ranging from 0 (*never*) to 5 (*always*). The Global Severity Index (GSI), which represents the average rating of all items, ranges from 0–5, with higher scores indicating greater body uneasiness. In the present study, Cronbach’s α was 0.86 for the GSI, 0.89 for the Weight Phobia (WP) subscale, 0.83 for the Body Image Concerns (BIC) subscale, 0.88 for the Avoidance (A) subscale, 0.78 for the Compulsive Self-Monitoring (CSM) subscale, and 0.86 for the Depersonalization (D) subscale.

#### Eating Disorder Inventory-3 Referral Form

The Eating Disorder Inventory-3 Referral Form** (**EDI-3-RF) is an abbreviated version of the original EDI-3 (Garner, 2004), which is a widely used self-report questionnaire for assessing the presence and intensity of psychological traits or symptoms that are clinically relevant to eating disorders in clinical and non-clinical populations. The present study considered the EDI-3-RF Body Dissatisfaction (BD) subscale, which consists of 10 items that explore discontentment with overall body shape and the size of regions of the body that are typically of significant concern to those with eating disorders (i.e., stomach, hips, thighs, buttocks; e.g., “I feel bloated after eating a normal meal”). Compared to previous versions of the scale, the EDI-3-RF has a six-choice format, but scores are recalibrated to a 0–4 format to expand the range of summative scores and improve the psychometric properties with non-clinical populations. The measure has been shown to yield adequate convergent and discriminant validity (Clausen et al., 2011), and all scales included in the EDI-3-RF have been found to have good reliability indices (Garner, 2004). In the present study, Cronbach’s α for the EDI-3-RF Body Dissatisfaction subscale was 0.84.

#### Forms of Self-Criticizing/Attacking & Self-Reassuring Scale

The Forms of Self-Criticizing/Attacking & Self-Reassuring Scale (FSCRS; Gilbert et al., 2004) is a self-report measure of the tendency to be self-critical and/or self-attacking in response to setbacks or failure. In line with the aims of the present study, which related to problematic attitudes towards one’s body, and in line with studies finding an association between both self-criticizing and self-attacking behaviors and shame related to body image, the present study employed two of the three FSCRS subscales: (a) Inadequate Self, which is comprised of 9 items measuring feelings of personal inadequacy and deficiency (e.g., “I am easily disappointed with myself”); and (b) Hated Self, which is comprised of 5 items that describe self-hating and self-harming behaviors, including those that are directed at the body (e.g., “I have become so angry with myself that I want to hurt or injure myself”). Items are rated on a 5-point Likert scale ranging from 0 (*not at all like me*) to 4 (*extremely like me*). The FSCRS has been shown to have good psychometric properties (Baiᾶo et al., 2015), and it has been previously applied to sexual minority populations (e.g., Petrocchi et al., 2020). In the present study, Cronbach’s α was 0.87 for the Inadequate Self subscale and 0.82 for the Hated Self subscale.

#### Demographic Information

The survey included a questionnaire that collected demographic data relating to age, assigned gender at birth (i.e., female, male), gender identity (i.e., cisgender woman, cisgender man, transgender woman, transgender man, other), ethnicity, education level, and sexual identity. Respondents indicated their sexual identity by selecting from one of five response options (1 = gay, 2 = lesbian, 3 = bisexual, 4 = heterosexual, 5 = other, please specify). They also provided their height and weight, for the calculation of BMI (measured as kg/m^2^).

## Data Analysis

### Power Analyses

A priori power analysis was conducted with G*Power version 3.1.9.7 (Faul et al., 2007), to determine the minimum sample size required for the analyses of principal interest. A multivariate analysis of variance (MANOVA) in the *F-*test family was run, with three groups and nine response variables. The input criteria were an error probability of .05, a conventional value of .95 as a threshold power to be reached, and a small effect size (*f*^*2*^) of .10 (Cohen, 1988). The findings showed that the projected minimum sample size was *N* = 156 participants.

### Data Analytic Plan

All statistical analyses were performed using the Statistical Package for the Social Sciences (SPSS Version 25) for Windows. First, group differences (in terms of assigned gender at birth and sexual identity) on the BUT-A and FSCRS subscales were analyzed using multivariate analyses of variance (MANOVAs). Subsequently, a one-way analysis of variance (ANOVA) was conducted to compare mean body dissatisfaction scores between LGB groups. Furthermore, partial eta squares for effect sizes were calculated. According to Cohen’s (1988) guidelines, partial η^2^ ≥ 0.01 was treated as a small effect, partial η^2^ ≥ 0.06 was regarded as a medium effect, and partial η^2^ ≥ 0.14 was considered a large effect. The Bonferroni test was applied for post-hoc comparisons. Next, hierarchical multiple regressions were conducted to investigate the relevance of BMI and body weight for overall body uneasiness, body dissatisfaction, and self-criticism amongst LGB subgroups, while controlling for participants’ age, assigned gender at birth, and sexual identity. Change in *R*^2^ was used to measure the significance of each step (i.e., block). The *F* test (i.e., *F*-change) was used to test whether *R*^2^ improvement was statistically significant. Partial correlations (i.e., partial *r*) were also reported to indicate the unique variance in the outcome variable predicted by each independent variable. There was no missing data at the item level, due to the survey’s forced item response.

## Results

### Comparisons Between Gay, Bisexual, and Heterosexual Men

Table 2 presents the results of the multivariate and univariate analyses of variance and the related effect sizes in the subgroups of gay, bisexual, and heterosexual men. A significant effect for sexual identity was detected for the BUT-A GSI and subscales (Wilks’ lambda = .87; *F*[2, 533] = 7.52; p < .001, η_p_^2^ = .07), as well as for the FSCRS subscales (Wilks’ lambda = .68, *F*[2, 533] = 19.91, p < .001, η_p_^2^ = .22). Significant differences also emerged for EDI-3-RF Body Dissatisfaction (*F*[2, 533] = 8.29, *p* < .001, η_p_^2^ = .04). Post-hoc comparisons indicated that, compared to heterosexual participants, gay and bisexual men scored significantly higher on the BUT-A GSI and subscales, and EDI-3-RF Body Dissatisfaction.

**Table 2:** Differences in Body Image Disturbances between Gay, Bisexual, and Heterosexual Men (*N* = 535)

Variable	Gay men (*n* = 277) *M (SD)*	Bisexual men (*n* = 39) *M (SD)*	Heterosexual men (*n* = 219) *M (SD)*	*F*	*p*	η^2^	Post-hoc
BUT-A^a^							
Weight Phobia	1.98 (0.96)	2.16 (1.09)	1.13 (0.91)	36.37	< .001	.12	G = BM > HM
Body Image Concerns	1.72 (1.02)	1.90 (1.28)	1.04 (0.99)	22.83	< .001	.08	G = BM > HM
Compulsive Self-Monitoring	1.17 (0.89)	1.32 (0.78)	0.71 (0.68)	18.49	< .001	.07	G = BM > HM
Avoidance	0.68 (0.67)	0.95 (0.68)	0.32 (0.59)	16.45	< .001	.06	G = BM > HM
Depersonalization	0.75 (0.90)	0.93 (0.99)	0.35 (0.53)	18.68	< .001	.07	G = BM > HM
Global Symptomatic Index	1.34 (0.98)	1.54 (1.11)	0.76 (0.68)	30.49	< .001	.10	G = BM > HM
EDI-3-RF^b^							
Body Dissatisfaction	11.37 (5.92)	10.72 (5.71)	8.32 (5.45)	8.29	< .001	.04	G = BM > HM
FSCRS^c^							
Inadequate Self	4.48 (0.50)	4.16 (0.65)	3.37 (0.50)	43.16	< .001	.16	G > BM > HM
Hated Self	4.88 (0.70)	4.36 (0.87)	3.56 (0.47)	45.98	< .001	.18	G > BM > HM

*G *gay men, *BM* bisexual men, *HM* heterosexual men

^a^Body Uneasiness Scale – A (Cuzzolaro et al., 2006)

^b^Eating Disorder Inventory-3 Referral Form (Garner, 2004)

^c^Forms of Self-Criticizing/Attacking & Self-Reassuring Scale (Gilbert et al., 2004)

Gay men also showed greater feelings of inadequacy and self-hate based on FSCRS subscales compared to bisexual men. Bisexual men, in turn, had higher levels of self-criticism than heterosexual men. The greatest effect sizes were found for FSCRS Inadequate Self and Hated Self, and BUT-A Weight Phobia, whereas the smallest effect size was found for EDI-3-RF Body Dissatisfaction.

### Comparisons Between Lesbian, Bisexual, and Heterosexual Women

Table 3 displays the results of the multivariate and univariate analyses of variance and the related effect sizes in the subgroups of lesbian, bisexual, and heterosexual women. Similar to the previous results, sexual identity had a significant effect for the BUT-A GSI and subscales (Wilks’ lambda = .94; *F*[2, 695] = 3.72; *p* < .001, η_p_^2^ = .03), as well as for the FSCRS subscales (Wilks’ lambda = .92, *F*[2, 695] = 4.87, *p* < .001, η_p_^2^ = .05); however, with lower effect sizes than those found in the subgroups of men. Conversely, no significant differences for EDI-3 Body Dissatisfaction were found (*F*[2, 695] = 1.36, *p* = ns, η_p_^2^ = .004). More specifically, post-hoc tests revealed that lesbian women scored lower on the BUT-A GSI, Weight Phobia, Body Image Concerns, and Compulsive Self-Monitoring, compared to bisexual and heterosexual women. In turn, bisexual women scored higher on BUT-A Avoidance and Depersonalization than lesbian and heterosexual women. Of note, the FSCRS results were mixed: lesbian women scored lower on Inadequate Self than bisexual women, and bisexual women scored lower on Inadequate Self than heterosexual women; however, lesbian women scored higher on Hated Self than bisexual women, and bisexual women scored higher on Hated Self than heterosexual women. All BUT-A effect sizes were small, whereas all FSCRS effect sizes were medium-large.

**Table 3:** Differences in Body Image Disturbances between Lesbian, Bisexual, and Heterosexual Women (*N* = 696)

Variable	Lesbian women (*N* = 163) *M (SD)*	Bisexual women (*N* = 135) *M (SD)*	Heterosexual women (N = 398) *M (SD)*	*F*	*p*	η^2^	Post-hoc
BUT-A^a^							
Weight Phobia	1.94 (0.99)	2.44 (1.28)	2.38 (1.27)	7.89	< .001	.02	L < BW = HW
Body Image Concerns	1.70 (1.33)	2.16 (1.29)	1.95 (1.26)	4.99	.007	.01	L < BW = HW
Compulsive Self-Monitoring	1.15 (1.03)	1.60 (1.10)	1.40 (1.08)	6.35	.002	.02	L < BW = HW
Avoidance	0.83 (0.72)	1.11 (0.68)	0.85 (0.79)	3.43	.033	.01	BW > L = HW
Depersonalization	0.81 (0.98)	1.22 (1.08)	0.91 (0.83)	6.06	.002	.02	BW > L = HW
Global Symptomatic Index	1.37 (1.03)	1.79 (1.04)	1.59 (1.01)	6.37	.002	.02	L < BW = HM
EDI-3-RF^b^							
Body Dissatisfaction	15.15 (6.79)	16.53 (6.24)	16.70 (6.10)	1.36	.257	.004	
FSCRS^c^							
Inadequate Self	3.31 (0.58)	4.12 (0.67)	4.51 (0.61)	39.22	< .001	.14	L < BW < HW
Hated Self	4.98 (0.30)	4.77 (0.81)	3.48 (0.65)	42.32	< .001	.16	L > BW > HW

*L* lesbian women, *BW* bisexual women, *HW* heterosexual women

^a^Body Uneasiness Scale – A (Cuzzolaro et al., 2006)

^b^Eating Disorder Inventory-3 Referral Form (Garner, 2004)

^c^Forms of Self-Criticizing/Attacking & Self-Reassuring Scale (Gilbert et al., 2004)

### Differences Between LGB Subgroups

Table 4 displays the group differences between LGB subgroups. A 3 (sexual identity: lesbian, gay, bisexual) × 2 (assigned gender at birth: man, woman) MANOVA was conducted on body uneasiness (BUT-A GSI and subscales) and self-criticism (FSCRS subscales). The choice of a 3 × 2 design was due to the low number of bisexual men, who were then merged with bisexual women. The analysis revealed a significant effect for sexual identity (Wilks’ lambda = .96; *F*[2, 614] = 1.90; *p* = .04, η_p_^2^ = .02), but no significant effect for the assigned gender at birth (Wilks’ lambda = .99; *F*[2, 614] = .5; *p* = .75, η_p_^2^ = .004), for the BUT-A subscales. Similarly, a significant effect for sexual identity (Wilks’ lambda = .70; *F*[2, 614] = 18.6; *p* < .001, η_p_^2^ = .15), but no significant effect for assigned gender at birth (Wilks’ lambda = 0.98; *F*[2, 614] = .5; *p* = .69, η_p_^2^ = .004), was found for the FSCRS subscales.

**Table 4:** Differences in Body Image Disturbances among LGB Individuals (*N* = 614)

Variable	Gay men (*N* = 277) *M (SD)*	Lesbian women (*N* = 163) *M (SD)*	Bisexual individuals (MW) (*N* = 174) *M (SD)*	*F*	*p*	η^2^	Post-hoc
BUT-A^a^							
Weight Phobia	1.98 (0.96)	1.94 (0.99)	2.38 (1.09)	6.29	.002	.02	BMW > G = L
Body Image Concerns	1.72 (1.02)	1.70 (1.33)	2.11 (1.28)	5.81	.003	.02	BMW > G = L
Compulsive Self-Monitoring	1.17 (0.89)	1.15 (1.03)	1.54 (0.78)	8.61	< .001	.03	BMW > G = L
Avoidance	0.68 (0.67)	0.83 (0.72)	1.07 (0.68)	8.07	< .001	.03	BMW > G = L
Depersonalization	0.75 (0.90)	0.81 (0.98)	0.97 (0.99)	6.64	< .001	.03	BMW > G = L
Global Symptomatic Index	1.34 (0.98)	1.37 (1.03)	1.74 (1.06)	9.31	< .001	.03	BMW > G = L
EDI-3-RF^b^							
Body Dissatisfaction	11.37 (5.92)	15.15 (6.79)	15.41 (7.20)	11.99	< .001	.04	L = BMW > G
FSCRS^c^							
Inadequate Self	4.48 (0.50)	3.31 (0.58)	4.13 (0.65)	38.16	< .001	.13	G > BMW > L
Hated Self	4.88 (0.70)	4.98 (0.30)	4.69 (0.87)	35.16	< .001	.11	L > BMW > G

*G* gay men, *L* lesbian women, *BMW *merged bisexual men and women

^a^Body Uneasiness Scale – A (Cuzzolaro et al., 2006)

^b^Eating Disorder Inventory-3 Referral Form (Garner, 2004)

^c^Forms of Self-Criticizing/Attacking & Self-Reassuring Scale (Gilbert et al., 2004)

Significant differences also emerged with respect to EDI-3-RF Body Dissatisfaction (*F*[2, 614] = 11.99, < .001, η_p_^2^ = .04). Post-hoc comparisons showed that bisexual individuals scored higher on all BUT-A subscales and the GSI compared to lesbian and gay participants, but with small effect sizes. With respect to EDI-3-RF Body Dissatisfaction, lesbian and bisexual participants scored higher than gay men, with small effect sizes. Similar to previous analyses, the FSCRS findings were mixed: gay men scored higher on Inadequate Self than bisexual individuals, and bisexual individuals scored higher on Inadequate Self than lesbian women; however, lesbian women scored higher on Hated Self than bisexual individuals and gay men. The FSCRS effect sizes were medium.

### The Predictive Role of BMI and Body Weight Among Sexual Minority Subgroups

A series of four hierarchical multiple regression analyses were performed to assess the role of BMI and body weight in predicting overall body uneasiness (BUT-A GSI), EDI-3-RF Body Dissatisfaction, and FSCRS Self-Criticism in LGB individuals (see Table 5). Age was entered in the first step; assigned gender at birth (i.e., man, woman) and sexual identity (i.e., lesbian/gay, bisexual) were entered in the second step; and body weight and BMI were entered in the third and final step.

**Table 5:** Hierarchical Regression Analyses for BMI and Body Weight Predicting Body Uneasiness, Body Dissatisfaction, and Self-Criticism in LGB Subgroups (*N* = 614)

	*R*	*R*^*2*^	β	F-change (Model)	*p*	Partial *r*
Criterion variable: BUT-A GSI^a^						
Step 1	.224	.050		31.988	< .001	
Age			−0.224			−.230***
Step 2	.266	.071		6.606	.001	
Assigned gender (man = 0, woman = 1)			0.048			.008
Sexual orientation (bisexual = 0, gay/lesbian = 1)			−0.142			−.158***
Step 3	.426	.182		40.792	< .001	
Body weight			−0.172			−.072
BMI (kg/m^2^)			0.473			.208***
Criterion variable: EDI-3 BD^b^						
Step 1	.185	.034		21.500	< .001	
Age			−0.185			−.157***
Step 2	.258	.067		10.485	< .001	
Assigned gender (man = 0, woman = 1)			0.037			.052
Sexual orientation (bisexual = 0, gay/lesbian = 1)			−0.164			−.132***
Step 3	.491	.241		68.893	< .001	
Body weight			−0.083			−.036
BMI (kg/m^2^)			0.487			.221***
Criterion variable: FSCRS IS^c^						
Step 1	.141	.020		12.357	< .001	
Age			0.141			.002
Step 2	.614	.377		77.507	< .001	
Assigned gender (man = 0, woman = 1)			−0.473			−.396***
Sexual orientation (bisexual = 0, gay/lesbian = 1)			−0.334			−.386***
Step 3	.620	.379		3.513	.030	
Body weight			−0.014			−.007
BMI (kg/m^2^)			−0.073			−.038
Criterion variable: FSCRS IS^d^						
Step 1	.263	.069		26.782	< .001	
Age			−0.263			−.086*
Step 2	.625	.416		79.367	< .001	
Assigned gender (man = 0, woman = 1)			0.497			.428***
Sexual orientation (bisexual = 0, gay/lesbian = 1)			0.167			.132**
Step 3	.628	.417		1.198	.257	
Body weight			−0.005			−.002
BMI (kg/m^2^)			−0.043			−.018

^*^p ≤ .05; **p ≤ .01; *** *p* ≤ .001

^a^Body Uneasiness Scale – A, Global Symptomatic Index (Cuzzolaro et al., 2006)

^b^Eating Disorder Inventory-3 Referral Form, Body Dissatisfaction subscale (Garner, 2004)

^c^Forms of Self-Criticizing/Attacking & Self-Reassuring Scale, Inadequate Self and Hated Self subscales (Gilbert et al., 2004). Partial correlations reported for the third and last step, in which all predictors were included in the regression model

In the final step, younger age (partial *r* = −.23, *p* < .001, and partial *r* = −.16, *p* < .001, respectively), sexual identity (partial *r* = −.16, *p* < .001, and partial *r* = −.13, *p* = .001, respectively), and higher BMI (partial *r* = .21, *p* < .001, and partial *r* = .22, *p* <0.001, respectively) emerged as significant predictors of the BUT-A GSI and EDI-3-RF Body Dissatisfaction, whereas assigned gender and body weight showed no significant effect. The final model explained 18.2% of the variance in overall body uneasiness, as assessed by the BUT-A GSI, and 24.1% of the variance in overall body dissatisfaction, as evaluated by EDI-3-RF Body Dissatisfaction.

Furthermore, in the last step, only assigned gender (partial *r* = −.39, *p* < .001) and sexual identity (partial *r* = −.38, *p* < .001) emerged as negative predictors of FSCRS Inadequate Self. On the other hand, assigned gender (partial *r* = .43, *p* < .001) and sexual identity (partial *r* = .13, *p* = .001) emerged as positive predictors of FSCRS Hated Self. The final models explained 37.9% and 41.7% of the variance in FSCRS Inadequate and Hated Self, respectively.

## Discussion

In-depth knowledge of the impact of body dissatisfaction and uneasiness among LGB individuals may have critical implications for the prevention and treatment of eating disorders, dysfunctional eating behaviors, and psychological distress among these populations (Thompson, 2000). The present results confirmed the first hypothesis, as gay and bisexual men displayed greater body and weight dissatisfaction, avoidance, compulsive control behaviors, detachment and estrangement feelings towards their body, and body uneasiness relative to heterosexual individuals. These findings are aligned with previous research suggesting that LGB persons—especially gay men—are more likely to experience body image concerns and may be less accurate in their body weight estimations, compared to heterosexual men (Dahlenburg et al., 2020; Morrison et al., 2004; Peplau et al., 2009; Russel & Keel, 2002). The literature suggests that men who are dissatisfied with their body are more likely to report disordered eating patterns, poorer health-related quality of life, greater psychological distress, and lower self-esteem (Bergeron & Tylka, 2007; Tylka, 2011; Wilson et al., 2013). These findings seem particularly relevant to gay men, who have been found to report greater interest in body modification strategies, as well as more pronounced appearance-related pressure and social comparison (Frederick & Essayli, 2016).

Similar results emerged with respect to self-attitude measures, with gay men reporting significantly greater feelings of inadequacy and self-hate than their heterosexual counterparts. As Gilbert et al. (2012) suggested, self-criticism and self-hate are highly associated with shame (Lingiardi et al., 2017), and their dysfunctional effects in LGB persons may derive from related self-directed negative emotions, including anger, disgust, and contempt (Petrocchi et al., 2020), which may also be focused on physical appearance and body shape. Interestingly, gay men also reported higher levels of self-criticism and self-hate compared to bisexual men—who, in turn, reported higher levels of self-blame and self-attack than heterosexual men. Despite the paucity of studies devoted explicitly to the self-attitudes and bodily representations of bisexual men, these results seem to partially diverge from previous research showing no differences between gay and bisexual men (e.g., Bridge et al., 2019). This might be explained by the small sample of bisexual men in the present study (discussed further below).

Sociocultural and objectification theories (Brewster et al., 2014) may provide a potential interpretation of the differences between men, in that several studies have found associations between sexual objectification experiences, internalization of body ideals, body surveillance, body shame, and self-criticism in sexual minority men (e.g., Wiseman & Moradi, 2010). Although some authors have suggested that heterosexual men also internalize body ideals, and are thus potentially subject to self-objectification (Daniel & Bridges, 2010; Martins et al., 2007), most studies have reported that sexual minority men are significantly more affected by this internalization (Duggan & McCreary, 2004; Hobza et al., 2007; Martins et al., 2007; Matsumoto & Rodgers, 2020). Minority stress theory (Meyer, 2003) may offer an additional framework for understanding how sexual minority men experience their bodies within the social environment (Mason & Lewis, 2016; Siconolfi et al., 2016), as the experience and internalization of negative feelings, attitudes, beliefs, behaviors, and assumptions about one’s sexual identity may have relevant consequences for self-perception, at both the identity and the bodily level (Balsam, 2001; Herek, 2000; Meyer, 1995; Rostosky et al., 2007).

Concerning the second hypothesis, the results were mixed. In line with expectations, no group differences were found in body dissatisfaction, as measured by the EDI-3-RF. This result is aligned with Morrison et al.’s (2004) meta-analysis, which found a non-significant effect size for the comparison of heterosexual and lesbian women on body satisfaction. However, with respect to the BUT-A subscales, lesbian women scored lower on Weight Phobia, Body Shape and Weight Dissatisfaction, Estrangement Feelings, Compulsive Control Behaviors, and Overall Body Uneasiness than bisexual and heterosexual women. These findings support the meta-analysis of Dahlenburg et al. (2020), which found that heterosexual women reported more significant body image disturbances compared to lesbian women across all measures considered, even with small effect sizes. They also align with evidence suggesting that lesbian women tend to have fewer disordered eating symptoms than heterosexual women (Lakkis et al., 1999; Strong et al., 2000). The present findings for lesbian women may be explained by a combination of objectification (Fredrikson & Roberts, 1997) and protective (Brown, 1987) theories: whereas women might feel pressurized to meet unrealistic standards of attractiveness, the lesbian subculture’s characteristic rejection of heteronormative standards and ideals may provide some protection against body image disturbances (Beck, 2017), body dissatisfaction (Alvy, 2013; Polimeni et al., 2009), negative attitudes towards weight (Owens et al., 2003), and negative appearance pressure (Hazzard et al., 2019) amongst lesbian women.

Similar findings emerged for FSCRS Self-Criticism and Self-Blaming Attitudes, with lesbian women reporting lower feelings of inadequacy relative to bisexual women—who, in turn, reported lower feelings of inadequacy relative to heterosexual women. Some authors have suggested that lesbian women’s lower feelings of inadequacy may be explained by their generally higher BMIs and preference for curvier figures (Alvy, 2013). However, in the present study, lesbian women also reported higher levels of self-hate and self-attack than bisexual and heterosexual women. Previous research with lesbian women has found that sexual identity–based discrimination is associated with less social support from the family, which, in turn, is related to increased negative affect, social anxiety, and disordered eating symptoms (Bell et al., 2019; Mason et al., 2018). Furthermore, shame and self-hate—primarily related to internalized homophobia—have been shown to be central psychological characteristics of disordered eating symptoms and binge behaviors in lesbian and bisexual women (Bayer et al., 2017).

The present results partially confirmed the third hypothesis, showing that lesbian and bisexual women tended to have more body dissatisfaction than gay men, as well as higher levels of self-hate and self-attack. Dahlenburg et al. (2020) found that, relative to gay men, lesbian women were more prone to experiencing body image disorders and to perceive themselves as physically unattractive (Markey et al., 2017; Swami, 2009). Although there have been very few studies on this topic, the present findings suggest that, while lesbian women may experience fewer body image disturbances than heterosexual women, they may still be influenced by societal pressure to achieve a certain body shape, and subsequently (consciously or subconsciously) experience higher self-objectification and body dissatisfaction relative to their male counterparts.

Nevertheless, contrary to expectations, bisexual individuals, irrespective of their assigned gender at birth, reported more body uneasiness than lesbian women and gay men. Lesbian women and gay men, in turn, reported similar levels of body uneasiness. Studies have shown that bisexual individuals with a same-sex partner tend to report more body image concerns than their heterosexual counterparts (Austin et al., 2004; Hadland et al., 2014). Furthermore, research has found that bisexual people typically report poorer mental health than gay/lesbian people (Conron et al., 2010; Herek, 2007; Kerr et al., 2013; Pistella et al., 2016), probably due to their greater difficulty achieving community and social support, which may hinder the emergence of positive identity dimensions (Baiocco et al., 2018; Petrocchi et al., 2020), even at a bodily level. However, more research on body image in bisexual populations is needed to support these explanations.

Concerning the fourth and final hypothesis, the results showed that younger age, bisexual identity, and higher BMI were significant predictors of the BUT-A GSI and overall body dissatisfaction. Body weight showed no significant effect. These findings are aligned with previous research suggesting that body concerns and dissatisfaction are related to elevated BMI, especially in samples with a diagnosed eating disorder (e.g., Muzi et al., 2020, 2021; Stice & Shaw, 2002). They also support studies confirming BMI as a predictor of weight-related concerns and weight management behaviors, even in non-clinical populations (Markey & Markey, 2005). Despite these observations, the present results and previous findings on the role of BMI in LGB populations are mixed. In fact, contrary to expectations, BMI did not emerge as a significant predictor of self-blame and self-attack. For instance, Frederick and Essayli (2016) found that, although BMI was a strong predictor of body dissatisfaction in sexual minority men, it did not consistently moderate the association between sexual identity and body image. Furthermore, Alvy (2013) suggested that BMI may represent a significant confounding factor in studies of body image in LGB populations, due to differences in self-reported versus directly measured values of height and weight and higher rates of obesity among lesbian women (e.g., Boehmer et al., 2007; Richmond et al., 2012).

Despite these results, the present study suffered from several limitations. First, the design was cross-sectional in nature and restricted to heterosexual and LGB individuals. Although the sample size was adequate, variables related to ethnicity, culture, and nationality were not measured. This limitation is particularly relevant, given the potential effects of ethnic and cultural representations of body ideals, body discrimination, and body image within LGB subgroups (e.g., Deputy & Boehmer, 2014; Gonzales & Blashill, 2021). Moreover, in the present study, the subsample of bisexual men was significantly smaller than the other subsamples. Thus, future research should seek to improve the present investigation by including larger, representative samples—especially with regard to bisexual individuals—and applying more sophisticated measures of sexual orientation.

Third, the data were collected through online sampling techniques, using only self-report measures. This may have generated different results relative to a research design using traditional in-person methodology and a multi-informant approach. However, a recent meta-analysis showed that online-based samples provide comparable results to those generated through in-person methods, suggesting that these methodologies do not significantly differ (Walter et al., 2019). Another limitation pertains to the data collection period, which immediately proceeded the most severe phases of the SARS-CoV-2 pandemic. Several studies have shown the negative consequences of the pandemic and its associated restrictive measures on psychological well-being and mental health, including disordered eating symptoms (Pierce et al., 2020; Prati & Mancini, 2021; Wu et al., 2021). While future studies should consider the potential long-term effect of the SARS-CoV-2 pandemic on body image concerns and disordered eating symptoms in LGB populations, a recent meta-analysis suggested that the pandemic’s adverse effects on eating were highly heterogeneous and challenging to control in empirical research (Sideli et al., 2021). Finally, the present study focused on cisgender LGB populations in comparison with a cisgender heterosexual population. Future studies should also investigate body image, body image disturbances, and internalized stigma among gender minority populations in comparison with a cisgender population, as previous research has shown that transgender and other gender minority individuals may experience significant body image disorders and concerns (e.g., Diemer et al., 2015). Furthermore, the representativeness of other gender and sexual minorities (e.g., queer, pansexual, asexual) should be enhanced, to generate better insight into the issues faced by the broader LGBTQ + community and to limit the dominance of majority voices (often gay men) in empirical studies on this topic (Salvati & Koc, 2022). Future prevention-oriented studies should also include measures of internalized sexual stigma and homonegativity (e.g., Lingiardi et al., 2012), in line with research suggesting a relationship between internalized homophobia and body shame, body image disorders, disordered eating attitudes, and binge eating in sexual minority individuals (e.g., Bayer et al., 2017; Williamson & Spence, 2001).

## Social Policy Implications

In-depth investigations into body image and body image disturbances in LGB individuals may generate significant clinical implications for interventions to treat and prevent a wide range of adverse health consequences (Calzo et al., 2015; Pistella et al., 2019). This is particularly relevant, as sexual minorities are exposed to unique stressors (i.e., minority stress in its various forms) that inevitably impact health outcomes (Hatzenbuehler & Pachankis, 2016; Kelleher, 2009; Lingiardi & Capozzi, 2004). In addition to being significantly associated with the onset of eating disorders (e.g., Bell et al., 2019; Simone et al., 2020), body image concerns and body dissatisfaction have also been found to predict the risk of bullying victimization, social discrimination, and diminished quality of life (Kamody et al., 2020; Lingiardi et al., 2020; Meyer, 2012).

From a wider perspective, prejudices and stereotyping attitudes against LGBTQ + people, with respect to body image and shape, are still widespread, both outside and within the LGBTQ + community, leading to potentially harmful consequences (Salvati et al., 2020). For instance, some studies have found that perceived stigma towards one’s body and physical appearance in same-sex couples is associated with self-doubt, sexual dissatisfaction, disempowerment, and lifestyle changes (Goldsmith & Byers, 2016; Markey & Markey, 2014; Markey et al., 2017). Other potential consequences of body dissatisfaction and uneasiness in sexual minority individuals might include an increased use of dating apps (e.g., Breslow et al., 2020), more romantic rejection (Foster-Gimbel & Engeln, 2016), and less professional success (Carels et al., 2022). Untangling the specific nuances of body image concerns and body-related experiences in individuals with different sexual identities and orientations may lead to the development of tailored, person-centered resources to help sexual minority individuals feel more positive about their bodies (Dahlenburg et al., 2020).

## Data Availability

The datasets generated during and/or analysed during the current study are available from the corresponding author on reasonable request.

## Code Availability

All reasonable requests for code relevant to the analyses of this paper will be met.
